# A mill based instrument and software system for dissecting slide-mounted tissue that provides digital guidance and documentation

**DOI:** 10.1186/1472-6890-13-29

**Published:** 2013-11-05

**Authors:** Nils Adey, Dale Emery, Derek Bosh, Steven Callahan, John Schreiner, Yang Chen, Ann Greig, Katherine Geiersbach, Robert Parry

**Affiliations:** 1 AvanSci Bio, LLC, 1290 West 2320 South, Suite D, Salt Lake City, UT 84119-1476, USA; 2 Department of Pathology, ARUP Laboratories, University of Utah, 500 Chipeta Way, Salt Lake City, UT 84108, USA; 3 SCI Institute, University of Utah, 72 S. Central Campus Dr., Room 3750, Salt Lake City, UT 84112, USA; 4 Department of Physiology, University of Utah, 420 Chipeta Way, Suite 1700, Salt Lake City, UT 84108, USA

**Keywords:** Slide-mounted tissue dissection, Microdissection, Mesodissection, Macrodissection, Digital pathology, Anatomic pathology tracking, FISH

## Abstract

**Background:**

Dissection of specific Areas Of Interest (AOIs) of slide-mounted tumor samples is often used to enrich for cancer cells in order to generate better signal to noise ratios in subsequent biochemical characterization. Most clinical laboratories utilize manual dissection for practical reasons and to avoid the expense and difficulties of laser microdissection systems. Unfortunately, manual methods often lack resolution and process documentation. The goal of this project was to design a dissection system for slide-mounted tissue with better precision than manual methods that also provides digital image guidance and electronic process documentation.

**Methods:**

An instrument that is essentially a micro tissue mill was developed. It employs a specialized disposable mill bit that simultaneously dispenses liquid, cuts tissue from the slide surface, and aspirates the liquid along with the displaced tissue fragments. A software package was also developed that is capable of transferring digitally annotated AOIs between images of serially cut tissue sections to guide dissection and generate an electronic record of the process.

**Results:**

The performance of this “meso” dissection system was tested using post dissection visual examination for resolution and accuracy, fluorescence based DNA quantitation for recovery efficiency, and dissection of closely situated mouse-human tissue sections followed by PCR amplification for purity determination. The minimum resolution is a dissected circle smaller than 200 microns in diameter, edge dissection accuracy is tighter than 100 microns, recovery efficiency appears greater than 95%, and recovery purity is greater than 99% relative to a different tissue located 100 microns from the dissection boundary. The system can dissect from both paraffinized and deparaffinized FFPE tissue sections that are mounted on plain glass slides, and it is compatible with DNA, RNA, and protein isolation.

**Conclusions:**

The mesodissection system is an effective alternative to manual dissection methods and is applicable for biomarker analysis of anatomical pathology samples, where enrichment of AOIs from the tissue section is helpful, but pure cell populations are not required.

## Background

Microscopic examination of formalin fixed, paraffin embedded (FFPE) tissue sections mounted on glass slides is a cornerstone of clinical histopathology. Often, ancillary molecular testing of these samples is required for further diagnosis, risk stratification, and treatment planning, particularly for cancer samples. This molecular testing typically involves mutation or expression analysis of nucleic acids or protein recovered from FFPE tissue sections. Due to the tissue heterogeneity of most slide-mounted tissue sections, regions enriched for the cell types of interest are often dissected directly off these tissue sections. In one of the most commonly used clinical techniques, an Area Of Interest (AOI) is hand annotated by a pathologist on an H&E stained, cover slipped, slide-mounted tissue section. This section is then sent to a laboratory where a technician manually aligns and traces the AOI onto the back of a second non-cover slipped slide containing a serially cut tissue section from the same tissue block. Manual macrodissection is then performed on the second slide using a scalpel or razor blade [1-3]. The process of identifying the AOI on an H&E stained and cover slipped slide and dissecting from a serial section allows for superior visualization of tissue morphology without the need to remove the coverslip, and dissection using a scalpel or razor blade is relatively quick and inexpensive. However, there are a number of drawbacks with this approach. Precision is significantly compromised due to the collective inaccuracy of the initial hand notation of the AOI, the thickness of the marking pen line, the tracing process, and the subsequent hand dissection such that the borders of the actual dissected region can vary by greater than +/- 1 mm from the borders of the region initially identified by the pathologist. This can be a significant problem when the area to be dissected is just a few millimeters wide. The process also tends to be poorly documented as the AOI and dissection notes are often hand written on the slide or accompanying paperwork. Moreover, the process typically requires the slides to be transported to multiple locations, and it is common practice to first remove the tissue surrounding the AOI in order to more efficiently recover the AOI with the straight edge of a razor or scalpel, leaving no record of the actual area dissected.

Two common alternatives to manual macrodissection that provide improved precision are manual microdissection and Laser MicroDissection (LMD). Manual microdissection has been used for decades [4,5] and is typically performed with a dissecting microscope and a hand held device such as needle or scalpel, which typically limits accuracy to +/- 200 microns, or a micromanipulator, where recovery of small groups of cells can be achieved [6-9]. Manual microdissection is tedious and time consuming, particularly when using micromanipulators, tissue retrieval on a needle can be tricky and unreliable, and process documentation is suboptimal [10]. Also, because it is difficult to physically annotate the areas to dissect with accuracy better than a few hundred microns, higher precision dissections require the operator to have training in order to select the appropriate cells. Alternatively, LMD instruments have been available for over a decade and have become highly sophisticated, providing automation, single cell resolution, and digital annotation and documentation. While LMD systems have many capabilities, they are very expensive and complex to operate, and many systems require the tissue sections to be mounted on special slides. If the areas to be dissected are indicated on a serial section, the variability between serial tissue sections will also limit the absolute precision.

A number of reports have investigated the level of dissection precision required for a variety of downstream assays [4,11-17]. The current consensus is manual macrodissection is adequate when the area to be dissected is at least a few millimeters in diameter and when the downstream assays are sufficiently sensitive to detect a mutant allele population of at least 20%. This is often the case for tests that assay specific genomic loci such as KRAS codon 12 and 13, and common variants in EGFR and BRAF, using technologies such as qPCR and pyrosequencing. Expression assays and more complex mutational assays typically benefit by some degree of microdissection, and while the precision of laser dissection is generally not necessary for most clinical assays [4,12,14,15], it can have significant benefits for discovery focused research studies [16,17]. Unfortunately, analysis of the degree of manual microdissection precision achieved by many of these comparison studies is difficult because the process is usually not well documented. The above information along with statements from many clinicians and researchers suggests there is a need for a relatively low cost dissection instrument with better precision than manual methods that also provides digital guidance and documentation of the dissection process. This report describes a novel instrumentation and software system designed to meet these needs. Such a system could improve clinical laboratory workflow, documentation, and help standardize clinical dissection precision requirements and terminology.

## Methods

### Study design, setting, participants, and methods

The development of the mesodissection system followed an ISO 9001 compliant phase review process. The breadboard prototype instrument was developed at AvanSci Bio using a modified commercial milling machine and the consumables were constructed from common hardware components and pipet tips. The laboratory prototype instrument and software were developed at AvanSci Bio, ARUP Laboratories, and the Scientific Computing and Imaging (SCI) Institute at the University of Utah, using a combination of custom and off the shelf components. Production consumables were manufactured from custom injection molded components. The imaging software was written in C++ and interfaces with various hardware drivers. The initial laboratory prototype was presented in the exhibit hall of the Association of Molecular Pathology 2011 Annual Meeting to obtain potential user feedback. This feedback was used to refine the design of the current instrument.

### Milling instrument and xScisor disposable

**Figure 1:**
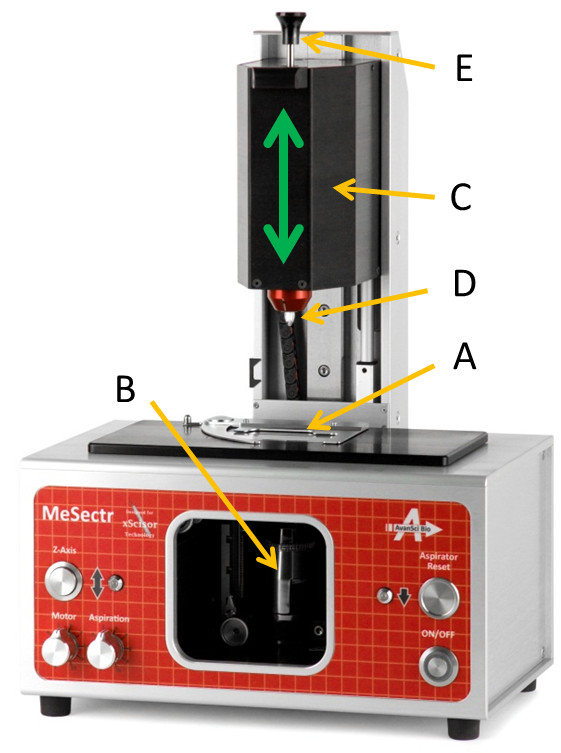
**The instrument.** The mesodissection instrument is essentially a modified tissue milling machine comprised of a joystick-driven X-Y slide stage **(A)**, a digital microscope **(B)** looking up through the slide adjustable from 5X to 60X total magnification, and a mill head **(C)** on a vertical axis mounted above the slide which lowers a spinning blade onto the slide surface to dissect the tissue. The blade is part of the disposable xScisor (**D**, described in detail in Figure 3), which simultaneously dispenses and aspirates fluid in a stage speed dependent rate in order to collect the tissue fragments. The user controls tissue dissection via a joystick while viewing the image from the digital microscope on a computer screen. Dissection can be guided using a digital outline that moves on the screen as the stage moves (See Figure 4). After the dissection is complete, the fragments are ejected into a small tube using the plunger control rod **(E)**.

**Figure 2:**
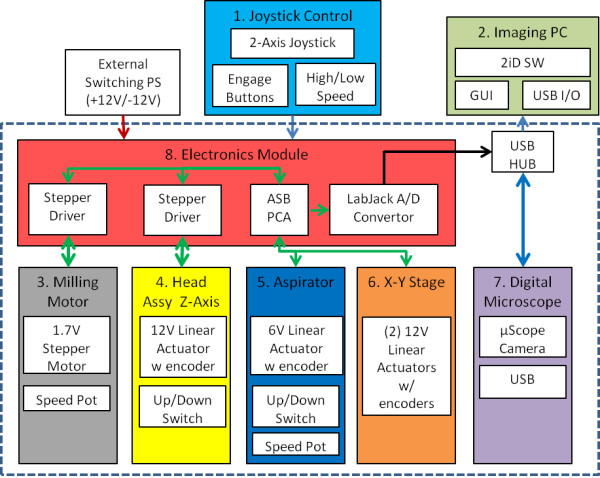
**Instrument architecture.** The instrument architecture is separated into eight major modules based on analog controls. **(1)** The 2-axis hall-effect joystick controls stage movement, milling motor rotation, Z axis head assembly position, and aspirator activation. **(2)** The core of the imaging software is a modified DNVideo-X application from AnMo Electronics that utilizes discreet instrument outputs via a LabJack A/D converter. **(3)** A hollow shafted stepper motor rotates the xScisor at a speed controlled by a separate user controlled potentiometer. **(4)** The Z Axis head assembly position is controlled by a linear actuator with an integrated potentiometer to enable 3 positions: load/unload, ready and contact. Contact position involves the linear actuator lowering the head assembly onto a height adjustable spring such that the downward force on the xScisor blade is minimized. **(5)** The instrument controls the xScisor fluid flow rate by withdrawing the xScisor plunger using a second linear actuator driven by the sum of X and Y axis absolute voltage inputs from the joystick, and an optional joystick pushbutton pulse control. **(6)** These joystick derived voltages also control the X and Y axis stage position through gear DC motors connected to cogged belt drives to pairs of parallel leadscrew drives. Stage positional feedback is received from a pair of 10 turn precision analog potentiometers via the A/D convertor and a shared USB multiplex plug. **(7)** The digital microscope is a 1.3 megapixel Dino-Lite by AnMo electronics connected to the imaging software via a shared USB multiplex plug. **(8)** The electronics module consists of the main circuit board, two driver boards, and the LabJack A/D converter.

**Figure 3:**
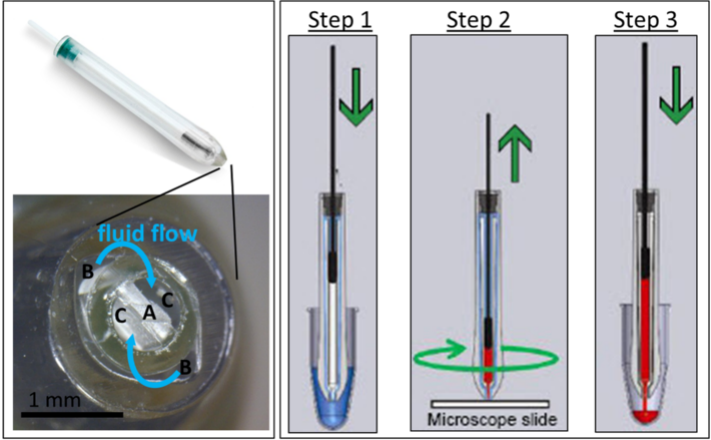
**The xScisor.** The xScisor is essentially a modified syringe consisting of two reservoirs, one above and one below the plunger, and a cutting blade that contacts the slide surface. The xScisor assembly consists of four parts: an outer syringe barrel that mates with the collet on the instrument head assembly, an inner syringe barrel containing the cutting blade **(A)** at one end, a plunger, and a plug that seals the plunger to the outer syringe body. The width of the dissected area is controlled by the width of the cutting blade; xScisors have been produced with 100, 200, 400, 800, and 1200 micron blades. To use the xScisor, the upper reservoir is filled with milling solution by repeatedly depressing and withdrawing the plunger (Step 1), and then it is loaded into the collet. The collet rotates the entire xScisor as it lowers it onto the slide surface (Step 2). As the stage is moved, the tissue section is driven into the rotating cutting blade and the plunger is withdrawn at a stage speed dependent rate. This action simultaneously dispenses liquid from the outer ports **(B)**, dissects the tissue, and aspirates the liquid along with the displaced tissue fragments into the inner ports **(C)**. A fully loaded xScisor can dispense up to 70 μl of milling solution; it is not necessary to use it all, only what is required to dissect the desired area. When dissection is complete, the head assembly is raised and the aspirated liquid containing the tissue fragments is collected by depressing the plunger (Step 3). The recovered tissue fragments can be pelleted by centrifugation and excess milling solution removed with a pipet. The xScisor can be either reloaded or discarded to avoid sample cross contamination.

A new platform that uses milling technology to dissect tissue from slide-mounted tissue sections was developed (See Figures 1 and 2 for a detailed description). In the basic mill design, an object is attached to a table (stage) capable of controlled X and Y axis movement such that it can be driven against a fixed rotating cutting bit thereby shaping the object. However, as milling is typically not used for the purpose of collecting fragments, a plastic mill bit termed the xScisor was developed that simultaneously dispenses liquid, cuts tissue, and aspirates the tissue fragments from the surface of the glass slide (Figure 3). Low cost manufacturing methods were developed so the xScisor could be disposable to prevent sample cross-contamination. Because tissue is relatively soft compared to glass, a spring pressure controlled system was designed such that the xScisor blade rests on the slide surface with sufficient downward force to cut through the tissue but glides across the glass slide. Milling tissue from glass slides also provided the opportunity to place a digital microscope below the slide in order to view the process, direct the dissection, and generate digital documentation.

### Software and workflow

**Figure 4:**
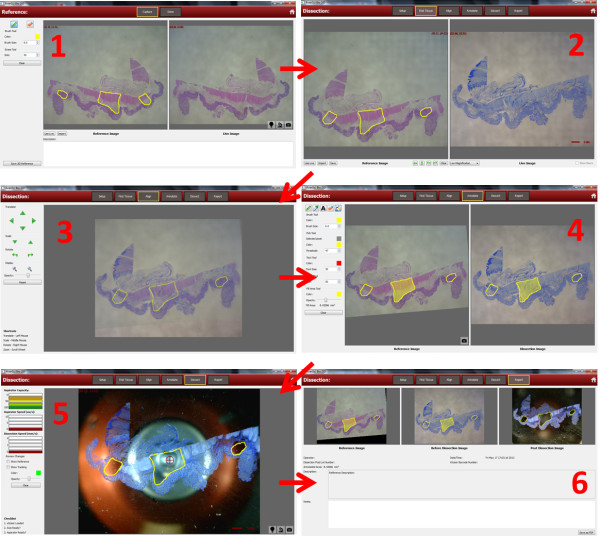
**Software and workflow.** The following description illustrates how the mesodissection 2iD software can assist the slide-mounted tissue dissection workflow used in many molecular pathology labs. **(1)** A pathologist would save a digital picture of a slide-mounted tissue section and then while viewing the tissue section in a standard optical microscope, digitally indicate the AOIs, then save this reference image to a digital database. Alternatively, digital scanning technology can generate the digital image, the AOIs marked, and the reference image saved in either .jpg, .png, or .tif format. To perform the dissection, **(2)** the reference image is imported from the database and manipulated to match the live image from the slide stage (on right) of a serial tissue section (cut from the same tissue block). **(3)** The reference image and a picture of the live image are digitally overlaid and the position of the reference image is further manipulated to achieve microscopic alignment. **(4)** The AOI(s) are selected using color based recognition and transferred to overlay the live image. It is also possible to directly annotate the live image and determine the area of each AOI. **(5)** As the stage is moved, the digital AOIs move in unison with the live image such that the digital AOIs can be used to guide the dissection process. A gauge indicates remaining xScisor capacity. Proper AOI movement is based on proper calibration of stage movement with the magnification of the digital image. **(6)** After dissection, a digital report is generated that includes the reference image, pre and post dissected images with AOI, and notes of the process.

A software package was developed that was modeled after a typical manual slide-mounted tissue dissection workflow found in many molecular pathology labs (discussed above, see Figure 4 for a more detailed description). This software provides an interface to digitally indicate AOIs and save dissection reference images. Alternatively, the dissection reference image can be generated by digital scanning technology if it is saved in either .jpg, .png. or .tif file format. In the laboratory, the reference image was imported from the database, aligned to the magnified live view from a serial tissue section, the AOI transferred to overlay the live image and guide the dissection, and finally a digital report in PDF format was generated.

### Tissue samples used in this study

All human FFPE tissue sections used for the studies described here were derived from left over/unused normal regions of placenta, liver, bowel, kidney, and skin specimens from the Anatomic Pathology Gross Room at the University of Utah (Department of Pathology, School of Medicine). As these tissue sections were anonymized, they are exempt from IRB approval. All human tissues were processed using standard FFPE methods, and the resulting tissue blocks and the mouse-human fusion tissue blocks were sectioned at 5 micron thickness. The formalin perfused mouse liver and kidney tissues that were used to make the mouse-human fusion blocks were surplus tissues obtained from studies of the mouse olfactory system done at the University of Utah (Department of Physiology). All the mouse olfactory tissue sections were from the same source, but had been OCT embedded, and then frozen sectioned at 10 to 60 microns thickness. These olfactory tissue sections were surplus from the intended studies. For the studies described here, the OCT was replaced with paraffin as described below.

### Resolution and accuracy

**Figure 5:**
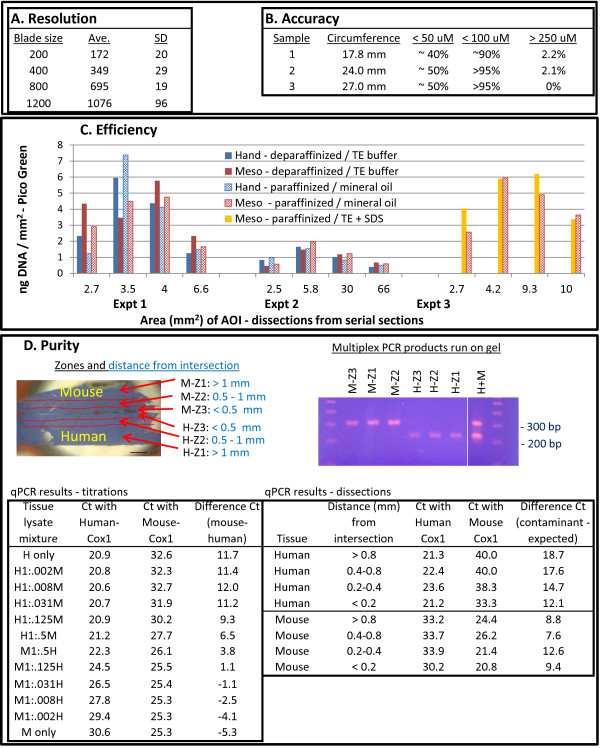
**Performance metrics. (A)** Resolution is defined as the width of the minimum dissectible area (a circle) generated by touching the rotating blade to the tissue without transverse movement (known as a point dissection). Each value is from five tests on each of five xScisors where the xScisor was removed and replaced into the collet after every dissection. Ave. = average, SD = Standard Deviation. **(B)** Accuracy is defined as the average distance between the intended boundary (using the digital overlay) and the actual boundary of dissection. The values shown are the percent of the linear distance around the circumference of the actual dissection boundary that was within 50 and 100 microns, or outside of 250 microns of the intended dissection boundary. This experiment was performed on a total of 14 AOIs from three different human liver tissue section samples. For each sample, the total circumference of the AOIs is shown. **(C)** Efficiency is measured by DNA quantitation using Pico Green following Proteinase K digestion; both mesodissection and manual dissection appear to be near 100% by visualization. Paraffinized tissue is efficiently aspirated using mineral oil, somewhat less efficiently using buffers containing SDS (but visualization is better), and not efficiently held in suspension by many aqueous buffers. **(D)** Purity is the percent of the recovered sample that is from the dissected area (a measure of potential contamination from adjacent undissected tissue). Purity was determined by dissecting immediately adjacent human and mouse 5 micron liver tissue sections at the indicated distances from the intersection, then testing by multiplex PCR containing one human and one mouse amplicon, or single amplicon qPCR as described in Results.

Both resolution and accuracy were quantitated using Microsoft PowerPoint by visual examination of the post-dissection digital images including the scale bars generated by the mesodissection software. The accuracy values were determined by attempting to fill the difference between the intended and actual boundary with a line of relative width of 50, 100, or 250 microns (Figure 5A and B). Standard deviation was calculated using Microsoft Excel.

### Efficiency

Serially cut tissue sections were either deparaffinized with AvanSci Bio’s mineral oil/alcohol system, or left paraffinized, then dissected by hand or using the mesodissection system. In order to dissect the same AOIs using both methods, the post manual dissected tissue sections were digitally outlined and used as guides for mesodissection. The resulting tissue samples were recovered in one of the following liquids: TE (10 mM Tris, 1 mM EDTA, pH 8.5), TE + 0.1% SDS, or light mineral oil, then subject to Proteinase K digestion by adding 5 μg of Proteinase K to the samples in TE and TE + SDS, or by adding 70 μl of TE with 5 μg proteinase K to the samples in mineral oil. Incubation was performed on the Thor (AvanSci Bio) programmable heater-shaker: 65°C 30 min 1500 rpm; 95°C 10 min 1500 rpm; 25°C 1 min 450 rpm. The samples were then centrifuged and assayed for genomic DNA concentration using PicoGreen according to the manufactures’ protocol (Life Technologies) as a measure of total sample recovered.

### Purity

To make the mouse-human fusion blocks, the formalin treated tissue samples described above were held in close proximity while casting in paraffin. The resulting 5 micron slide-mounted tissue sections were deparaffinized and dissected as indicated. The recovered tissue samples were Proteinase K digested as described above, then subject to both dual amplicon multiplex endpoint PCR with Taq Platinum using the recommended conditions (Life Technologies) and 2% agarose gel electrophoresis, or single amplicon qPCR using the Power SYBR Green master mix and the recommended conditions (Life Technologies). The PCR primer pair (amplicons) sequences (directed to either the mouse or human Cox1 mitochondrial genes) are: AGGGGACCCAATTCTCTACCA + CTCCGTGTAGGGTTGCAAGT (mouse) and TTCGGCGCATGAGCTGGAGTCC + AGTTGCCAAAGCCTCCGATT (human).

### RNA isolation, reverse transcription, and quantitative PCR

The formalin perfused, OCT embedded, frozen sectioned, 10 to 60 microns thick mouse olfactory tissue sections that were obtained for this study were relatively poorly adhered to the slides surface and tended to be dislodged rather than cut by the spinning xScisor blade. Therefore, the tissue sections were gently rinsed in 0.1X PBS to remove the OCT, dipped in molten paraffin for 5 minutes, then the excess paraffin allowed to drain by standing the slide on edge at 70°C for 5 minutes. These tissue sections were milled using 70 μl per sample of light mineral oil, then 70 μl of TE, pH 8.5 containing 5 μg of protease K was added and the tubes mixed. Protease K digestion was carried out using the Thor heater-shaker at 60°C 30 min 1500 rpm; 82°C 15 min 1500 rpm; 25°C 1 min 450 rpm. The overlaying mineral oil was removed using Wicking Strips (AvanSci Bio), then 90 μl PKD buffer (Qiagen) was added to achieve 160 μl total volume. Next, 16 μl of DNase booster buffer and 10 μl of DNase I (Qiagen) were added to each tube, the tubes incubated at room temperature for 15 minutes and the RNA isolated as described in the RNeasy FFPE kit (Qiagen). The manual dissected material was processed using the limonene deparaffinization protocol described in the RNeasy FFPE kit. cDNA was generated using the Applied BioSystems High Capacity Reverse Transcription kit and Quantitative PCR was performed using Applied BioSystems Power SYBR Green reagent as described in the manufacturer’s protocol. The primers pairs used in the qPCR were GATGACTGAGTACCTGAACCG + CAGAGACAGCCAGGAGAAATC (mouse Bcl-2 cDNA) and GCCCTCCGTATCTTACTTCAAG + GCGGTCCAGGTAGTTCATG (mouse Cyclin D1 cDNA).

### FISH

Slide-mounted tissue sections were deparaffinized using d-limonene or AvanSci Bio’s mineral oil/alcohol system, then mesodissected using TE plus 0.1% Tween-20 as the milling solution. Recovered tissue fragments were centrifuged for 2 minutes, the majority of the supernatant discarded, the fragments resuspended, and 2 μl aliquots were spotted onto Fisher Scientific Capillary Gap plus slides (130 micron) and baked at 65°C for 2 hours. These slides were then subject to tissue FISH processing using the Kreatech recommended FFPE Tissue FISH protocol.

## Results

### Mesodissection capabilities and performance metrics

Four performance metrics were established to test the mesodissection technology: resolution, accuracy, efficiency, and purity. The resolution (Figure 5A) is the minimum dissectible area determined using point dissections and is primarily dependent on the diameter of the cutting blade but is also dependent on any non-concentricity of the blade (runout). Currently, the minimum resolution is a circle about 170 microns in diameter. xScisors with 100 micron plastic cutting blades have been made but the wear rate was excessive; a steel tip blade is currently under development. A second metric, accuracy (Figure 5B), is a measure of how well the user can guide stage movement using the joystick (transverse movement). The average accuracy from this test, performed by a single user, was 60 microns. Most users with practice can achieve 100 micron accuracy, and point dissection accuracy is often better as the user can precisely position the blade before lowering it onto the slide surface. The third metric, recovery efficiency (Figure 5C), is determined by Pico Green quantitation of DNA following Proteinase K digestion of the recovered tissue. The recovery efficiency of both mesodissection and manual macrodissection are similar and also appear to be near 100% by visualization. The fourth metric, purity (Figure 5D) is a measure of potential contamination from neighboring undissected tissue (for example, if DNA were to leach out of the surrounding tissue and be picked up by the milling solution). Purity was determined by dissecting immediately adjacent human and mouse 5 micron liver tissue sections at variable distances from the intersection. The recovered samples were treated with Proteinase K, then subjected to multiplex endpoint PCR/gel electrophoresis, and single amplicon quantitative PCR. The PCR primer pairs (amplicons) were directed to either the human or mouse Cox1 mitochondrial sequence. As seen in the gel image, while both bands are present from a control containing an equal amount of each tissue (H + M), only the expected band is detectable from the dissected samples indicating little to no contaminating template. For the qPCR titration experiments, the entire section of human and mouse tissue were dissected separately, treated with Proteinase K, and then mixed at the ratios shown. While the human Cox1 amplicon amplified earlier than the mouse, the response to relative template ratios is clearly visible. In the single sample dissections, very little contaminating template (the titration experiments indicate it is significantly less than 1%) is recovered even from dissections immediately adjacent to the tissue intersection. The results from both multiplex endpoint PCR and real time qPCR indicate purity is greater than 99%, but this result could potentially be sample dependent.

### Applications

**Figure 6:**
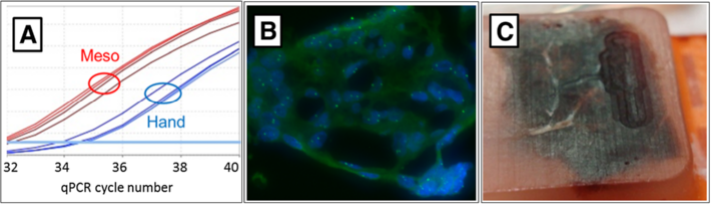
**Applications. (A)** qPCR analysis (using a mouse cyclin D1 gene amplicon) of cDNA prepared from RNA isolated from paraffinized FFPE mouse olfactory tissue. The same AOIs from serial tissue sections were either mesodissected using mineral oil, or manual (hand) dissected where the recovered tissue was deparaffinized in limonene and recovered by centrifugation (loss during the centrifugation steps probably account for the lower yield of the manual protocol). Two samples were isolated using each method and two qPCR reactions performed for each sample. Similar results were obtained using a mouse Bcl-2 gene amplicon (not shown). **(B)** FISH imaged fragments of tissue sections. FFPE tissue sections from human placenta (not shown: liver, bowel, and kidney performed similarly) were deparaffinized, mesodissected, and re-adhered to charged glass slides. These slides were processed using standard tissue FISH protocols and assayed with a chromosome 17 centromeric probe, then stained with DAPI. **(C)** Paraffinized tissue block milled with the mesodissection instrument.

The mesodissection system was applied to two common techniques in molecular diagnostics, RNA expression analysis and anatomical FISH (Fluorescent In Situ Hybridization) analysis in order to investigate capability. Because RNA is labile, many labs prefer to leave the tissue sections paraffinized during dissection to minimize exposure to moisture and atmospheric oxygen. Therefore, mineral oil was used as the milling solution, as it can aspirate paraffinized tissue and maintain a non-aqueous environment (used in Figure 5C). The recovered mineral oil/tissue fragment mixture was added directly to an equal volume of aqueous Proteinase K reaction solution where it formed a separate upper phase. When the tube was subject to heating and shaking, the tissue fragments migrated to the lower aqueous phase and were digested by the Proteinase K, which also digests RNase. Following the digestion, the organic phase was removed and RNA isolation, cDNA synthesis and quantitative PCR were performed using commercial kits. The results indicate the mesodissected material produced a larger quantity of RNA, as assayed by Reverse Transcription-quantitative Polymerase Chain Reaction (RT-qPCR), but sample loss during the additional centrifugation steps used for deparaffinization of the manual dissected sample likely accounts for the difference (Figure 6A). A link to a poster describing an endpoint RT-PCR analysis of these same samples is available at http://www.avanscibio.com.

Tissue fragments that had been mesodissected from slide-mounted tissue sections were re-adhered to slides and used for FISH analysis (Figure 6B). The majority of the fragments were present in a single thickness (not folded or stacked) with minimal obvious damage. The tissue fragments remained bound to the slides throughout the subsequent FISH processing steps and produced good quality FISH chromosomal signals. Testing using a variety of human tissue samples (described above, all 5 microns thick) and dissection conditions found that fragment size (determined by visual inspection using a scale bar) was variable ranging from just a few cells to over a millimeter in diameter. However, for a given dissection condition, over 90% of the total tissue area was within a 2-3 fold size range. Dissection of well adhered tissue (for example paraffinized tissue dissected using mineral oil) produced small fragments (2-10 cells in diameter) whereas dissection of less well adhered tissue (for example deparaffinized pre-moistened tissue) produced larger fragments (half to one millimeter in diameter). No obvious tissue type specific effects on tissue fragment size were noted, but we did observe that the tissue tended to tear along natural boundaries. For example, cross sections of kidney tubules remained intact as small rings of cells. We also noted some physical dissection and handling effects on fragment size. For example, larger slower rotating xScisor blades and gentle use of pipet tips with larger openings in the subsequent handling steps tended to produce larger tissue fragments.

An FFPE tissue block was mounted onto the mesodissection instrument stage using tape and the Z-axis contact position of the xScisor adjusted using potentiometers on the electronics board (an earlier version instrument). The tissue block was then dissected using mineral oil and overhead visual guidance (Figure 6C). The depth of the cut appeared to be related to the depth of the blade as the end of the inner syringe barrel appeared to ride on or very near the upper surface of the tissue block. Tissue fragments along with the expected volume of mineral oil were recovered suggesting sufficient dissection liquid fluid flow exists, possibly through the path cut in the tissue block. A significant problem with this approach is difficulty visualizing the dissection process in real time; the tissue block is opaque making visualization from beneath the block very difficult.

## Discussion

Dissection of specific Areas Of Interest (AOIs) directly from slide-mounted tissue sections is commonly used to enrich for cell types of interest for further molecular analysis. Most clinical labs utilize manual dissection methods, typically of tumor samples, for cost and simplicity reasons. The only other viable choice for most clinical needs is Laser MicroDissection (LMD), which is extremely precise but also costly and often complicated. Because of this, a number of clinicians have expressed interest in a more automated, precise, and digitally documented approach to slide-mounted tissue dissection. This interest will likely grow as complex tests involving molecular techniques such as expression analysis, next generation sequencing, and proteomics are increasingly utilized. In cases where the downstream biochemistry is expensive, it may also become cost effective to improve the quality of the input sample. This interest lead to the development of the mesodissection platform described here. We use the term “meso” because the precision is between that of LMD and manual macrodissection methods. The current version mesodissection system provides better than 200 micron resolution and the joystick control allows users to obtain accuracy approaching +/- 60 microns from the intended region. In our experience, this is better than most users can obtain using a dissection microscope and scalpel. However, since the precision of both mesodissection and manual dissection are operator dependent, it is difficult to quantitate the degree of precision improvement. The mesodissection software allows a user (such as a pathologist) to make AOI decisions using an optical image, then annotate these AOIs on a digital image. Use of digital images can eliminate the need for hand annotated slides to be sent to the dissection lab thus minimizing logistical issues. In the laboratory, the serial tissue sections are digitally aligned, the AOIs electronically transferred to the live images, and used as a dissection guide. We find that the use of digital microscopic annotation typically results in more precision than hand annotation. Finally, the software generates a digital report of the entire dissection process.

### Biochemical considerations

The mesodissection system is compatible with both paraffinized and deparaffinized FFPE tissue sections mounted on standard glass slides. A variety of milling solutions have been tested and many more presumably can be used as long as they do not degrade the plastic xScisor and can hold the tissue fragments in suspension. For the latter reason mineral oil or SDS containing solutions were used to hold paraffinized fragments in suspension. When using mineral oil, the subsequent Proteinase K reaction can be performed by adding an aqueous solution to the recovered fragments and incubating on a heater-shaker. In these conditions, the tissue fragments migrate from the upper organic phase into the lower aqueous phase where the digestion occurs immediately. For expression analysis studies, dissection of still paraffinized tissue using organic milling solution is desirable as it minimizes exposure of the tissue to air and moisture prior to the Proteinase K step. A problem with the use of mineral oil is dissection visualization is somewhat impaired. Dissection visualization of paraffinized tissue sections is better using solutions containing SDS. SDS solutions can aspirate paraffinized tissue to a modest degree and SDS is compatible with Proteinase K. However, SDS must be removed prior to most subsequent biochemical reactions whereas post Proteinase K tissue lysates recovered from mineral oil can go directly into many downstream biochemistries.

Every type of transparent slide tested including both plain and charged glass was compatible with mesodissection as long as the tissue sections were reasonably well-adhered to the surface. Standard FFPE sections of most tissue types and thicknesses worked well, but occasional difficulty was encountered dissecting calcified tissue (the blade tends to ride over the top of the tissue). We found that frozen sectioned OCT embedded tissue was sometimes poorly adhered to the glass surface and when contacted by aqueous milling solution tended to dislodge rather than dissect. The problem tends to be worse with thicker sections (greater than 10 microns). In these cases, rinsing with an aqueous buffer to remove the OCT, dehydrating in alcohol, then dipping in molten paraffin and allowing to drain at an elevated temperature in order to remove the majority of molten paraffin greatly improved adhesion and provided good quality RNA dissection. Partial deparaffinization using AvanSci Bio’s mineral oil/alcohol system allows for tissue staining and improved visualization.

We used the mesodissection system to generate samples for two common applications, RT-qPCR and FISH. The results show that mesodissected samples are compatible with RNA expression analysis using RT-qPCR. The recovered sample-mineral oil mixture can be added directly to a Proteinase K reaction without an extraction and centrifugation step. Elimination of this step likely improved the RNA yield compared to a commonly used limonene deparaffinization protocol. We demonstrate that fragments of tissue generated by mesodissection can be re-adhered to slides and used for FISH analysis. This application could be used to increase FISH throughput as well as minimize hybridization probe and precious sample usage. A collaborator has used the mesodissection system to obtain samples for protein analysis by mass spectrometry and reports the system is comparable in quality and yield to other methods of tissue sample retrieval (Dr. David Krizman, Oncoplex Diagnostics). We also showed that the mesodissection system is capable of dissection directly from tissue blocks. Dissection directly from tissue blocks produces a lot of sample since the cut depth approaches 100 microns without the need to generate and dissect individual slide-mounted tissue sections. A possible downside is that the deeper the dissection, the less information one has about the material being recovered. Our further development of this capability is dependent on user interest, as it will require engineering of clamps mounted on the X-Y stage to properly position the tissue blocks and improvements to the dissection visualization methods.

### Factors improving dissection performance

In addition to the factors discussed above, the following list of milling variables can improve mesodissection performance and should be considered when operating the instrument: 1) The cutting effectiveness, diameter of the cut area, and wear rate of the blade is a function of the downward force of the mill head, controlled using the adjustable position spring stop. Once properly set, it is usually not necessary to readjust the spring pressure. However, sometimes it is necessary to increase pressure when using larger blades, particularly when cutting paraffinized tissue, and in this situation, going over the same area multiple times can be helpful. 2) Coordinated movement of the digital AOI with the live image of the tissue section is dependent on precise calibration of the instrument’s slide stage with the 2iD software. 3) The aspiration rate can affect recovery efficiency; in general, increase the aspiration rate to the point where most of the dissection solution is used for a given sample, then centrifuge to pellet the recovered sample and discard excess dissection solution. 4) When doing point dissections, it is necessary to depress the pulse button for a couple seconds in order to aspirate the dissected tissue, otherwise aspiration occurs only with transverse stage movement. 5) To obtain clean cut edges, the leading edge of the blade should be rotating into the leading edge of dissection. For example, because the blade rotates counter-clockwise, dissection around the outside edge of an AOI should proceed in a counter-clockwise direction. 6) When dissecting deparaffinized tissue with TE or water, the dissected tissue occasionally forms clumps, which can be flung outside of the aspiration area by the rotating xScisor rather than being aspirated. Addition of a small amount of detergent (i.e. 0.1% Tween-20) to the milling solution can greatly reduce the formation of tissue clumps and improve aspiration. 7) The design of the xScisor results in simultaneous dispense and aspirate functions because both functions are controlled by the same movement of the plunger. The aspiration volume is greater than the dispense volume by the volume of the xScisor plunger. The effect is the column of collected liquid is regularly broken by small air bubbles. If a small amount of milling liquid is pre-aspirated, these air bubbles can be used as boluses to physically separate dissected areas or place an air bolus between the plunger and the recovered liquid, which can be used to increase recovery efficiency. The disadvantage of pre-aspiration is a reduction of total aspiration capacity.

### Next generation instrument

While the current mesodissection instrument provides a number of advantages related to the digital workflow, it is joystick driven and thus typically does not result in a time savings compared to hand dissection of larger AOI’s. A number of laboratories have expressed interest in automation and therefore, we are developing the next generation instrument with a fully automated stage. The AOIs will be transferred manually as this step requires user verification. However, once transferred, the software will automatically design a milling path for each AOI and perform the dissection at a significantly faster rate. This additional capability not only frees user from dissecting using the joystick, but minimizes any impact of impaired dissection visualization when using unstained and still paraffin embedded tissues.

## Conclusions

A mill based instrument system with a sample cutting and collecting consumable and digital guidance and documentation software was developed for dissecting slide-mounted tissue. The system is capable of 200 micron resolution, better than 100 micron accuracy, and greater than 95% recovery efficiency. This mesodissection system can improve dissection precision and documentation compared to the manual dissection and annotation methods currently used in most clinical laboratories. This instrumentation system is useful for isolating anatomical pathology samples for subsequent analysis of components such as DNA, RNA, and proteins, particularly when enrichment of specific regions on the tissue section is helpful, but pure cell populations are not required.

## Abbreviations

AOI: Area of interest; LMD: Laser micro dissection.

## Competing interests

NA, DE, DB, and RP are employees of, and YC is a consultant for AvanSci Bio, LLC that currently markets the mesodissection system, the topic of this manuscript. NA and RP are also members and managers of AvanSci Bio, LLC. SC, JS, AG, and KG are employees of the University of Utah, which holds a minor share in AvanSci Bio, LLC. AvanSci Bio and xScisor are registered trademarks of AvanSci Bio, LLC. Patent applications are pending on various aspects of this technology.

## Authors’ contributions

NA conceived and built the breadboard prototype instrument and xScisor, conducted and oversaw most of the biochemical characterization, contributed to subsequent design of the instrument, xScisor, and software, and wrote the manuscript. DE executed much of the instrument design and contributed to xScisor design. DB contributed to design, characterization, and manufacture scale up of the xScisor and instrument. SC and JS helped conceive and coded all of the 2iD software. YC helped design and conducted the RNA based studies, and ran mesodissection experiments. AG provided assistance with the RT-qPCR experiments, generated the mouse tissue and olfactory tissue sections, ran mesodissection experiments, and provided manuscript review. KG helped conceive and specify the requirements for the initial system design. RP helped conceive, design, and build the laboratory prototype instrument and xScisor, contributed subsequent design of the instrument, xScisor, and software, and oversaw the entire system design and manufacture scale-up. All authors read and approved the final manuscript.

## Pre-publication history

The pre-publication history for this paper can be accessed here:

http://www.biomedcentral.com/1472-6890/13/29/prepub

## References

[B1] Kim HK Kim J Korolevich S Choi IJ Kim CH Munroe DJ Green JE Distinctions in gastric cancer gene expression signatures derived from laser capture microdissection versus histologic macrodissection BMC Med Gen 2011 4 48 10.1186/1755-8794-4-48 PMC3141377 21635755

[B2] Kovalev S Marchenko ND Gugliotta BG Chalas E Chumas J Moll UM Loss of p53 function in uterine papillary serous carcinoma Hum Pathol 1998 29 613 619 10.1016/S0046-8177(98)80012-9 9635683

[B3] Brink M de Goeij AFPM Weijenberg MP Roemen GMJM Lentjes MHFM Pachen MMM Smits KM de Bruïne AP Goldbohm RA van den Brandt PA K-ras oncogene mutations in sporadic colorectal cancer in The Netherlands Cohort Study Carcinogenesis 2003 24 703 710 10.1093/carcin/bgg009 12727799

[B4] Hernández S Lloreta J Manual versus laser micro-dissection in molecular biology Ultrastruct Pathol 2006 30 3 221 228 10.1080/01913120500521018 16825124

[B5] Going J Histological microdissection in diagnostic and investigative pathology Diagn Histopathol 2009 16 1 43 48

[B6] Beltinger CP Debatin KM A simple combined microdissection and aspiration device for the rapid procurement of single cells from clinical peripheral blood smears Mol Pathol 1998 51 4 233 236 10.1136/mp.51.4.233 9893754 PMC395645

[B7] Nzula S Going JJ Stott DI Antigen-driven clonal proliferation, somatic hypermutation, and selection of B lymphocytes infiltrating human ductal breast carcinomas Cancer Res 2003 63 12 3275 3280 12810659

[B8] Lee J Dong S Kim S Yoo N Lee S Park W A simple, precise and economical microdissection technique for analysis of genomic DNA from archival tissue sections Virchows Arch 1998 433 305 309 10.1007/s004280050253 9808431

[B9] Going J Lamb R Practical histological microdissection for PCR analysis J Pathol 1996 179 121 124 10.1002/(SICI)1096-9896(199605)179:1<121::AID-PATH536>3.0.CO;2-D 8691336

[B10] Bova GS Optimal molecular profiling of tissue and tissue components: defining the best processing and microdissection methods for biomedical applications Methods Mol Med 2005 103 15 66 15542897

[B11] Martin DN Boersma BJ Yi M Reimers M Howe TM Yfantis HG Tsai YC Williams EH Lee DH Stephens RM Weissman AM Ambs S Differences in the tumor microenvironment between African-American and European-American breast cancer patients PLoS One 2009 4 2 e4531 Epub 10.1371/journal.pone.0004531 19225562 PMC2638012

[B12] Moelans C de Weger R Ezendam C van Diest P HER-2/neu amplification testing in breast cancer by Multiplex Ligation-dependent Probe Amplification: influence of manual- and laser microdissection BMC Cancer 2009 9 4 10.1186/1471-2407-9-4 19123950 PMC2631004

[B13] Williams C Pontén F Moberg C Söderkvist P Uhlén M Pontén J Sitbon G Lundeberg J A high frequency of sequence alterations is due to formalin fixation of archival specimens Am J Pathol 1999 155 5 1467 1471 10.1016/S0002-9440(10)65461-2 10550302 PMC1866966

[B14] de Bruin EC van de Pas S Lips EH van Eijk R van der Zee MM Lombaerts M van Wezel T Marijnen CA van Krieken JH Medema JP van de Velde CJ Eilers PH Peltenburg LT Macrodissection versus microdissection of rectal carcinoma: minor influence of stroma cells to tumor cell gene expression profiles BMC Genom 2005 6 142 10.1186/1471-2164-6-142 PMC1283972 16225673

[B15] El-Serag HB Nurgalieva ZZ Mistretta TA Finegold MJ Souza R Hilsenbeck S Shaw C Darlington G Gene expression in Barrett’s esophagus: laser capture vs. whole tissue Scand J Gastroenterol 2009 44 787 793 10.1080/00365520902898127 19391063 PMC2822542

[B16] Klee EW Erdogan S Tillmans L Kosari F Sun Z Wigle DA Yan P Aubry MC Vasmatzis G Impact of sample acquisition and linear amplification on gene expression profiling of lung adenocarcinoma: laser capture micro-dissection cell-sampling versus bulk tissue-sampling BMC Med Genom 2009 2 13 10.1186/1755-8794-2-13 PMC2667433 19272143

[B17] Michel C Desdouets C Sacre-Salem B Gautier JC Roberts R Boitier E Liver gene expression profiles of rats treated with clofibric acid. Comparison of whole liver and laser capture microdissected liver Am J Pathol 2003 163 2191 2199 10.1016/S0002-9440(10)63577-8 14633594 PMC1892366

